# Crystal structure of *fac*-aquatricarbonyl[(*S*)-valin­ato-κ^2^ *N*,*O*]­rhenium(I)

**DOI:** 10.1107/S2056989016005235

**Published:** 2016-03-31

**Authors:** Kseniia O. Piletska, Kostiantyn V. Domasevitch, Alexander V. Shtemenko

**Affiliations:** a Department of Inorganic Chemistry, Ukrainian State University of Chemical Technology, Gagarin Ave. 8, Dnipropetrovsk 49005, Ukraine; b Inorganic Chemistry Department, National Taras Shevchenko University of Kyiv, Volodymyrska Street 64/13, Kyiv 01601, Ukraine

**Keywords:** crystal structure, rhenium carbonyl complex, valine

## Abstract

In the mol­ecule of the title compound, [Re(C_5_H_10_NO_2_)(CO)_3_(H_2_O)], the Re^I^ atom adopts a distorted octa­hedral coordination sphere defined by one aqua and three carbonyl ligands as well as one amino N and one carboxyl­ate O atom of the chelating valinate anion. The carbonyl ligands are arranged in a *fac*-configuration around the Re^I^ ion. In the crystal, an intricate hydrogen-bonding system under participation of two O—H, two N—H and one C—H donor groups and the carboxyl­ate and carbonyl O atoms as acceptor groups contribute to the formation of a three-dimensional supra­molecular network.

## Abstract 2

The title mol­ecule consists of an Re(CO)_3_^+^ fragment, an aqua ligand and one *N,O*-chelating valinate anion to complete a slightly distorted coordination sphere.

## Chemical context   

The syntheses of metal–organic compounds, which are capable of visualization of biomolecules, is receiving growing inter­est in biocoordination chemistry (Coogan & Fernández-Moreira, 2014 ▸). For the labeling of biomolecules, octa­hedral *fac*-tricarbonyl complexes of Tc and Re are the most promising compounds (Alberto, 2007 ▸; Coogan *et al.*, 2014 ▸). The compact *M*(CO)_3_-core (*M* = Tc, Re) allows labeling of low mol­ecular weight substrates under retention of activity and specificity. In this context, Re(CO)_3_^+^ compounds are of inter­est as the closest non-radioactive analogs of ^99m^Tc-based systems, which could be particularly important for visualization and immunotherapy. Studies of the cytotoxicity of rhenium carbonyl complexes also suggest their specific anti­cancer activity (Leonidova & Gasser, 2014 ▸).
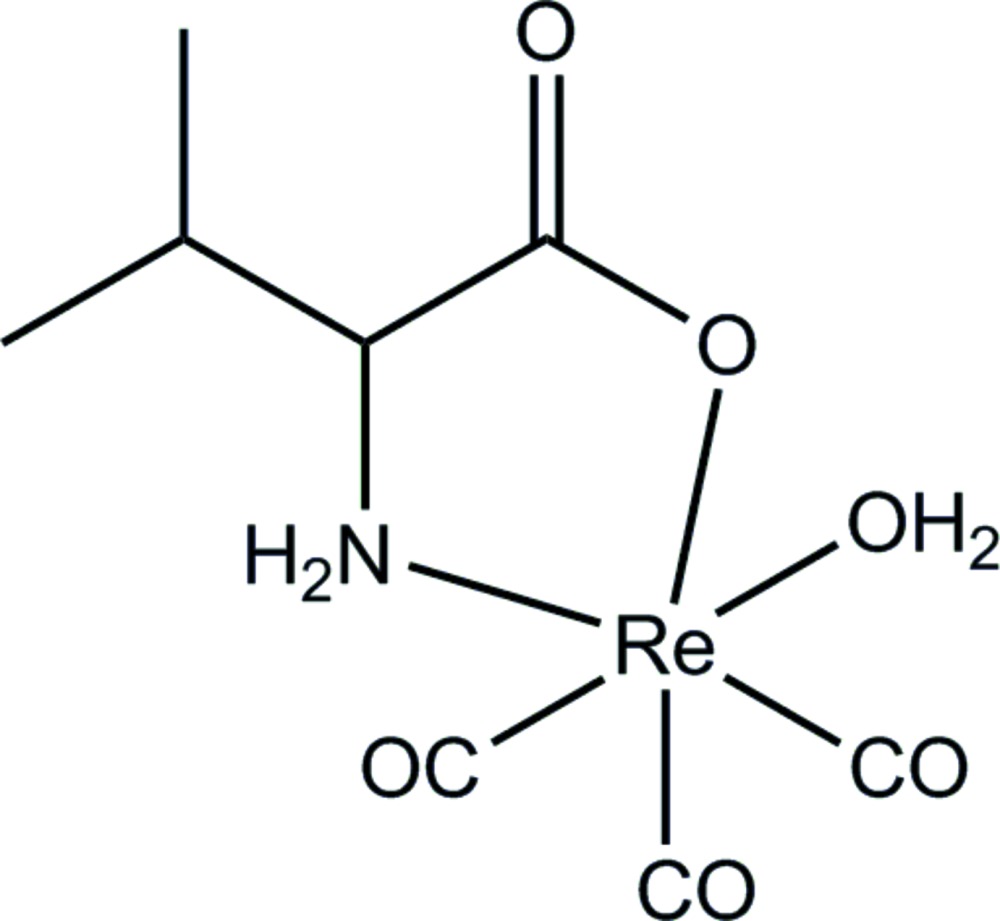


Most of the known Re(CO)_3_^+^ complexes with biologically essential substrates comprise tridentate co-ligands, *e.g*. histidinato-*O,N,N′* (Alberto *et al.*, 1999 ▸), me­thio­ninato-*N,O,S* (He *et al.*, 2005 ▸), 2,3-di­amino­propionato-*N,N′,O* (Liu *et al.*, 2006 ▸), completing the coordination octa­hedra of the central ions. At the same time, coordinatively unsaturated complexes of bidentate amino­carboxyl­ates could be suited for inter­actions with additional ligands, such as guanine bases (Zobi *et al.* 2005*a* ▸), thus allowing an attractive scenario for the assembly of mixed-ligand systems.

In this communication, we report the synthesis and crystal structure of a novel Re(CO)_3_^+^ complex with valine and water as co-ligands. Following the findings of Zobi *et al.* (2005*b* ▸), sufficient reactivity of this compound towards DNA may be anti­cipated.

## Structural commentary   

**Figure 1:**
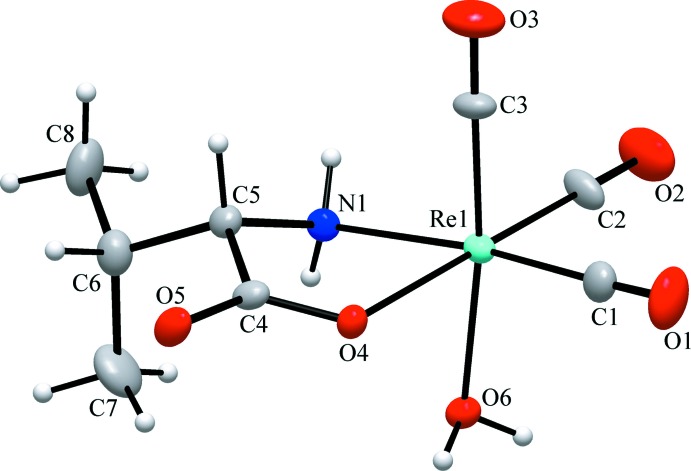
The mol­ecular structure of the title complex, with displacement ellipsoids drawn at the 40% probability level.

In the mol­ecule of the title compound (Fig. 1 ▸), the Re^1^ ion resides in a slightly distorted octa­hedral coordination environment, with a facial arrangement of three nearly equidistant carbonyl ligands [Re1—C bond lengths are in the range 1.881 (7)–1.909 (7) Å]. The compound crystallizes in the chiral space group *P*2_1_2_1_2_1_, with the *S*-enanti­omer of the valinate anion present in the selected crystal. The anion coordinates in a bidentate-chelating fashion through the amino N and one carboxyl­ate O atoms, with Re1—N1 and Re1—O4 bond lengths of 2.195 (5) and 2.122 (4) Å, respectively. The five-membered chelate ring [bite angle N1—Re1—O4 = 74.62 (18)°] has the expected envelope conformation, with the atoms of the Re1—O4—C4—C5 fragment being coplanar within 0.035 (3) Å and the N1 flap atom deviating from the given mean plane by 0.547 (6) Å. The Re1—O6 bond involving the aqua ligand [2.175 (5) Å] is slightly longer than the one with the carboxyl O atom. The CO ligands coordinate in an almost linear fashion, with O—C—Re bond angles spanning a range from 175.5 (7) to 179.9 (8)°, while the corresponding C—Re1—C angles are within 87.1 (3)–89.8 (2)°. All other bond length and angles are comparable to those found for related Re^I^ complexes (Rajendran *et al.*, 2000 ▸).

## Supra­molecular features   

**Figure 2:**
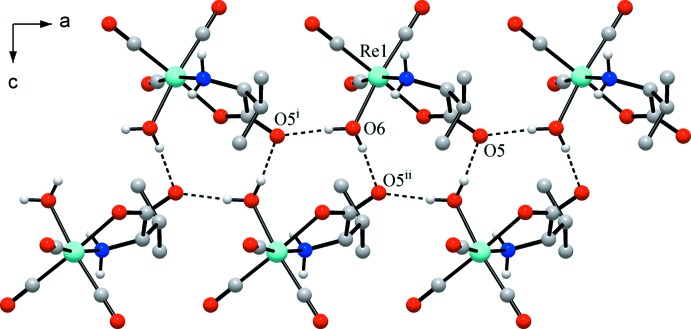
Primary supra­molecular inter­actions involving rather strong O—H⋯O bonds that produce chains parallel to the *a* axis. [Symmetry codes: (i) *x* − 1, *y*, *z*; (ii) *x* − 0.5, −*y* + 0.5, −*z* + 1.]

**Figure 3:**
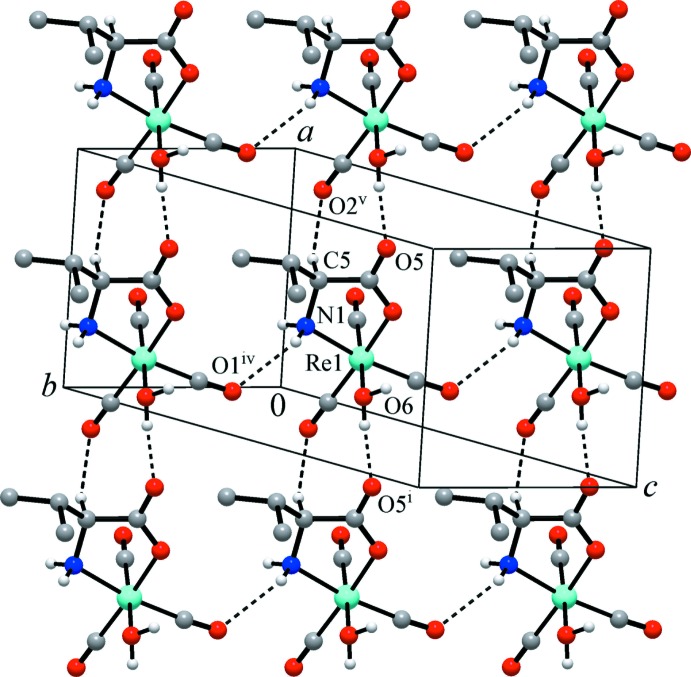
The crystal structure of the title complex showing all hydrogen-bonding inter­actions (O—H⋯O, N—H⋯O and C—H⋯O) as dashed lines. The isopropyl CH-hydrogen atoms were omitted for clarity. [Symmetry codes: (i) *x* − 1, *y*, *z*; (iv) *x*, *y* + 1, *z*; (v) *x* + 1, *y*, *z*.]

In the crystal, the packing of the mol­ecules is governed by an intricate system of hydrogen bonds, including classical O—H⋯O and N—H⋯O bonds and weaker C—H⋯O inter­actions (Table 1 ▸). Two rather strong and nearly linear O—H⋯O bonds are observed between the aqua ligand and the non-coordinating carboxyl­ate O atoms of two symmetry-related neighbouring mol­ecules. The amino group forms two weaker N—H⋯O bonds to carbonyl O atom acceptor groups of two neighbouring mol­ecules. Each non-coordinating carboxyl­ate O atom accepts two such bonds, yielding hydrogen-bonded chains parallel to the *a*-axis direction (Fig. 2 ▸), whereas the N—H⋯O bonds expand the hydrogen-bonding system into a three-dimensional network. Additional C—H⋯O inter­actions consolidate this arrangement (Fig. 3 ▸). The combination of O—H⋯O and C—H⋯O (involving the chiral C5 atom) bonds may be important for the observed enanti­oselective packing of the chiral moieties (Petkova *et al.*, 2001 ▸).

## Synthesis and crystallization   

To a solution of dl-valine (0.116 g, 0.984 mmol) in 5 ml of water, a solution of tri­aqua­tri­carbonyl­rhenium(I) bromide (0.100 g, 0.246 mmol) in 10 ml of methanol was added. The reaction mixture was heated and stirred at 343 K under a steady stream of argon for 4 h. After cooling to room temperature, the solution was left to evaporate in air for a period of a few days. After removal of the methanol co-solvent, a colourless crystalline product formed. The precipitate was collected by suction filtration, washed with water and then with a 50 ml portion of petroleum ether and dried (yield: 0.068 g, 68%). Suitable single crystals were obtained by slow diffusion of hexane vapor into a methanol solution of the complex. IR (KBr, cm^−1^): ν_as_(CO) 2028 (*s*), ν_s_(CO) 1905 (*s*).

## Refinement   

Crystal data, data collection and structure refinement details are summarized in Table 2 ▸. C-bound hydrogen atoms were placed geometrically and refined using a riding model, with C—H = 0.97 Å and *U*_iso_(H) = 1.5*U*_eq_(C) for methyl and with C—H = 0.99 Å and *U*_iso_(H) = 1.2*U*_eq_(C) for methine groups. N- and O-bound hydrogen atoms were found from difference maps and refined with N—H = 0.90 Å, O—H = 0.85 Å and *U*_iso_(H) = 1.2*U*_eq_(N,O).

## Supplementary Material

Crystal structure: contains datablock(s) I. DOI: 10.1107/S2056989016005235/wm5283sup1.cif

Structure factors: contains datablock(s) I. DOI: 10.1107/S2056989016005235/wm5283Isup2.hkl

CCDC reference: 1469075

Additional supporting information:  crystallographic information; 3D view; checkCIF report

## Figures and Tables

**Table 1:** Hydrogen-bond geometry (Å, °)

*D*—H⋯*A*	*D*—H	H⋯*A*	*D*⋯*A*	*D*—H⋯*A*
O6—H1*W*⋯O5^i^	0.85	1.85	2.693 (5)	175
O6—H2*W*⋯O5^ii^	0.85	1.88	2.723 (5)	175
N1—H1*N*⋯O3^iii^	0.90	2.15	2.979 (7)	153
N1—H2*N*⋯O1^iv^	0.90	2.41	3.103 (6)	133
C5—H5⋯O2^v^	0.99	2.59	3.527 (7)	158

Symmetry codes: (i) 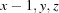; (ii) 
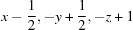
; (iii) 
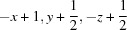
; (iv) 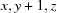; (v) 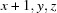.

**Table 2:** Experimental details

Crystal data	
Chemical formula	[Re(C_5_H_10_NO_2_)(CO)_3_(H_2_O)]
*M*_r_	404.39
Crystal system, space group	Orthorhombic, *P*2_1_2_1_2_1_
Temperature (K)	213
*a*, *b*, *c* (Å)	7.1229 (5), 7.2913 (7), 22.6098 (18)
*V* (Å^3^)	1174.24 (17)
*Z*	4
Radiation type	Mo *K*α
μ (mm^−1^)	10.36
Crystal size (mm)	0.16 × 0.12 × 0.12

Data collection	
Diffractometer	Stoe Imaging plate diffraction system
Absorption correction	Numerical (*X-SHAPE* and *X-RED*; Stoe, 2001 ▸)
*T*_min_, *T*_max_	0.288, 0.370
No. of measured, independent and observed [*I* > 2σ(*I*)] reflections	10442, 2809, 2546
*R*_int_	0.040
(sin θ/λ)_max_ (Å^−1^)	0.660

Refinement	
*R*[*F* ^2^ > 2σ(*F* ^2^)], *wR*(*F* ^2^), *S*	0.022, 0.047, 0.99
No. of reflections	2809
No. of parameters	147
H-atom treatment	H-atom parameters constrained
Δρ_max_, Δρ_min_ (e Å^−3^)	1.68, −0.91
Absolute structure	Flack *x* determined using 990 quotients [(*I* ^+^)−(*I* ^−^)]/[(*I* ^+^)+(*I* ^−^)] (Parsons *et al.*, 2013 ▸).
Absolute structure parameter	−0.018 (10)

Computer programs: *IPDS Software* (Stoe, 2000 ▸), *SHELXS97* (Sheldrick, 2008 ▸), *SHELXL2014/7* (Sheldrick, 2015 ▸), *DIAMOND* (Brandenburg, 1999 ▸) and *WinGX* (Farrugia, 2012 ▸).

## supplementary crystallographic information

### Crystal data

**Table:**

[Re(C_5_H_10_NO_2_)(CO)_3_(H_2_O)]	*D*_x_ = 2.287 Mg m^−^^3^
*M**_r_* = 404.39	Mo *K*α radiation, λ = 0.71073 Å
Orthorhombic, *P*2_1_2_1_2_1_	Cell parameters from 8000 reflections
*a* = 7.1229 (5) Å	θ = 2.9–28.0°
*b* = 7.2913 (7) Å	µ = 10.36 mm^−^^1^
*c* = 22.6098 (18) Å	*T* = 213 K
*V* = 1174.24 (17) Å^3^	Prism, colorless
*Z* = 4	0.16 × 0.12 × 0.12 mm
*F*(000) = 760	

### Data collection

**Table:**

Stoe Imaging plate diffraction system diffractometer	2546 reflections with *I* > 2σ(*I*)
Radiation source: fine-focus sealed tube	*R*_int_ = 0.040
φ oscillation scans	θ_max_ = 28.0°, θ_min_ = 2.9°
Absorption correction: numerical (*X-SHAPE* and *X-RED*; Stoe, 2001)	*h* = −9→9
*T*_min_ = 0.288, *T*_max_ = 0.370	*k* = −9→9
10442 measured reflections	*l* = −29→29
2809 independent reflections	

### Refinement

**Table:**

Refinement on *F*^2^	Secondary atom site location: difference Fourier map
Least-squares matrix: full	Hydrogen site location: mixed
*R*[*F*^2^ > 2σ(*F*^2^)] = 0.022	H-atom parameters constrained
*wR*(*F*^2^) = 0.047	*w* = 1/[σ^2^(*F*_o_^2^) + (0.0254*P*)^2^] where *P* = (*F*_o_^2^ + 2*F*_c_^2^)/3
*S* = 0.99	(Δ/σ)_max_ = 0.002
2809 reflections	Δρ_max_ = 1.68 e Å^−^^3^
147 parameters	Δρ_min_ = −0.91 e Å^−^^3^
0 restraints	Absolute structure: Flack *x* determined using 990 quotients [(*I*^+^)-(*I*^-^)]/[(*I*^+^)+(*I*^-^)] (Parsons *et al.*, 2013).
Primary atom site location: structure-invariant direct methods	Absolute structure parameter: −0.018 (10)

### Special details

**Table:**

Geometry. All e.s.d.'s (except the e.s.d. in the dihedral angle between two l.s. planes) are estimated using the full covariance matrix. The cell e.s.d.'s are taken into account individually in the estimation of e.s.d.'s in distances, angles and torsion angles; correlations between e.s.d.'s in cell parameters are only used when they are defined by crystal symmetry. An approximate (isotropic) treatment of cell e.s.d.'s is used for estimating e.s.d.'s involving l.s. planes.

### Fractional atomic coordinates and isotropic or equivalent isotropic displacement parameters (Å^2^)

**Table:**

	*x*	*y*	*z*	*U*_iso_*/*U*_eq_	
Re1	0.27215 (3)	0.24317 (4)	0.36414 (2)	0.01911 (8)	
O1	0.1402 (9)	−0.1539 (7)	0.3751 (3)	0.0475 (16)	
O2	−0.0548 (9)	0.3311 (10)	0.2820 (3)	0.059 (2)	
O3	0.4865 (10)	0.1103 (10)	0.2566 (3)	0.0510 (17)	
O4	0.4953 (6)	0.2146 (6)	0.42564 (19)	0.0201 (10)	
O5	0.7729 (7)	0.3107 (6)	0.4559 (2)	0.0296 (11)	
O6	0.1463 (7)	0.3556 (6)	0.4439 (2)	0.0216 (10)	
H1W	0.0276	0.3449	0.4455	0.032*	
H2W	0.1929	0.3046	0.4744	0.032*	
N1	0.4095 (7)	0.5125 (7)	0.3670 (3)	0.0202 (10)	
H1N	0.4012	0.5560	0.3298	0.030*	
H2N	0.3545	0.5944	0.3913	0.030*	
C1	0.1826 (10)	−0.0024 (10)	0.3716 (4)	0.0300 (15)	
C2	0.0674 (10)	0.2983 (10)	0.3127 (3)	0.0309 (18)	
C3	0.4063 (11)	0.1628 (11)	0.2973 (3)	0.0283 (16)	
C4	0.6284 (10)	0.3294 (9)	0.4248 (3)	0.0215 (14)	
C5	0.6095 (10)	0.4941 (9)	0.3839 (3)	0.0216 (14)	
H5	0.6814	0.4668	0.3475	0.026*	
C6	0.6917 (11)	0.6706 (9)	0.4109 (4)	0.0319 (16)	
H6	0.8235	0.6446	0.4220	0.038*	
C7	0.5880 (13)	0.7280 (13)	0.4668 (4)	0.048 (2)	
H7A	0.4638	0.7721	0.4565	0.072*	
H7B	0.6574	0.8250	0.4864	0.072*	
H7C	0.5768	0.6236	0.4931	0.072*	
C8	0.6942 (12)	0.8254 (9)	0.3654 (5)	0.0424 (19)	
H8A	0.5664	0.8610	0.3561	0.064*	
H8B	0.7566	0.7837	0.3298	0.064*	
H8C	0.7613	0.9298	0.3816	0.064*	

### Atomic displacement parameters (Å^2^)

**Table:**

	*U*^11^	*U*^22^	*U*^33^	*U*^12^	*U*^13^	*U*^23^
Re1	0.01711 (11)	0.02296 (11)	0.01726 (10)	0.00198 (16)	−0.00083 (9)	−0.00125 (15)
O1	0.035 (3)	0.027 (3)	0.081 (5)	−0.005 (2)	−0.001 (3)	0.002 (3)
O2	0.038 (4)	0.091 (5)	0.048 (4)	0.017 (3)	−0.016 (3)	−0.006 (3)
O3	0.060 (5)	0.061 (4)	0.032 (3)	0.016 (3)	0.012 (3)	−0.006 (3)
O4	0.018 (2)	0.020 (3)	0.023 (2)	−0.0010 (17)	−0.0010 (17)	0.0027 (17)
O5	0.015 (2)	0.043 (2)	0.030 (2)	0.0011 (19)	−0.005 (2)	0.0118 (19)
O6	0.019 (2)	0.024 (2)	0.022 (2)	0.0027 (18)	0.0015 (19)	0.0026 (18)
N1	0.020 (3)	0.020 (2)	0.020 (3)	0.0023 (19)	−0.002 (3)	0.003 (2)
C1	0.020 (4)	0.037 (4)	0.032 (4)	−0.002 (3)	−0.004 (3)	0.000 (3)
C2	0.023 (4)	0.043 (5)	0.026 (4)	0.011 (3)	−0.010 (3)	−0.010 (3)
C3	0.027 (4)	0.041 (4)	0.016 (3)	0.006 (3)	0.005 (3)	−0.001 (3)
C4	0.017 (4)	0.028 (3)	0.019 (3)	0.005 (3)	0.003 (3)	0.002 (2)
C5	0.017 (3)	0.025 (3)	0.023 (3)	0.002 (2)	0.000 (2)	0.000 (2)
C6	0.024 (4)	0.031 (3)	0.041 (4)	0.000 (3)	−0.005 (3)	−0.001 (3)
C7	0.055 (5)	0.039 (5)	0.050 (5)	0.005 (5)	−0.006 (4)	−0.022 (4)
C8	0.033 (5)	0.027 (3)	0.066 (6)	−0.007 (3)	−0.002 (5)	0.004 (4)

### Geometric parameters (Å, º)

**Table:**

Re1—C3	1.881 (7)	N1—H1N	0.9004
Re1—C2	1.908 (7)	N1—H2N	0.9004
Re1—C1	1.909 (7)	C4—C5	1.520 (9)
Re1—O4	2.122 (4)	C5—C6	1.539 (9)
Re1—O6	2.175 (5)	C5—H5	0.9900
Re1—N1	2.195 (5)	C6—C7	1.523 (11)
O1—C1	1.148 (9)	C6—C8	1.526 (11)
O2—C2	1.139 (9)	C6—H6	0.9900
O3—C3	1.148 (9)	C7—H7A	0.9700
O4—C4	1.265 (8)	C7—H7B	0.9700
O5—C4	1.255 (8)	C7—H7C	0.9700
O6—H1W	0.8498	C8—H8A	0.9700
O6—H2W	0.8503	C8—H8B	0.9700
N1—C5	1.482 (8)	C8—H8C	0.9700
			
C3—Re1—C2	88.0 (3)	O5—C4—O4	122.3 (6)
C3—Re1—C1	87.1 (3)	O5—C4—C5	119.9 (6)
C2—Re1—C1	89.8 (3)	O4—C4—C5	117.8 (6)
C3—Re1—O4	96.6 (3)	N1—C5—C4	108.3 (5)
C2—Re1—O4	173.0 (2)	N1—C5—C6	113.1 (5)
C1—Re1—O4	95.8 (3)	C4—C5—C6	112.8 (6)
C3—Re1—O6	173.2 (3)	N1—C5—H5	107.5
C2—Re1—O6	96.4 (3)	C4—C5—H5	107.5
C1—Re1—O6	98.2 (3)	C6—C5—H5	107.5
O4—Re1—O6	78.61 (18)	C7—C6—C8	111.2 (7)
C3—Re1—N1	94.4 (3)	C7—C6—C5	111.9 (6)
C2—Re1—N1	99.8 (3)	C8—C6—C5	110.9 (7)
C1—Re1—N1	170.4 (3)	C7—C6—H6	107.5
O4—Re1—N1	74.62 (18)	C8—C6—H6	107.5
O6—Re1—N1	79.7 (2)	C5—C6—H6	107.5
C4—O4—Re1	119.1 (4)	C6—C7—H7A	109.5
Re1—O6—H1W	114.3	C6—C7—H7B	109.5
Re1—O6—H2W	110.2	H7A—C7—H7B	109.5
H1W—O6—H2W	108.2	C6—C7—H7C	109.5
C5—N1—Re1	110.8 (4)	H7A—C7—H7C	109.5
C5—N1—H1N	109.6	H7B—C7—H7C	109.5
Re1—N1—H1N	105.0	C6—C8—H8A	109.5
C5—N1—H2N	108.7	C6—C8—H8B	109.5
Re1—N1—H2N	114.7	H8A—C8—H8B	109.5
H1N—N1—H2N	107.9	C6—C8—H8C	109.5
O1—C1—Re1	175.5 (7)	H8A—C8—H8C	109.5
O2—C2—Re1	179.9 (8)	H8B—C8—H8C	109.5
O3—C3—Re1	178.6 (7)		
			
Re1—O4—C4—O5	−173.0 (5)	O5—C4—C5—C6	−37.3 (9)
Re1—O4—C4—C5	6.6 (7)	O4—C4—C5—C6	143.1 (6)
Re1—N1—C5—C4	−31.1 (6)	N1—C5—C6—C7	60.3 (8)
Re1—N1—C5—C6	−156.7 (5)	C4—C5—C6—C7	−63.0 (8)
O5—C4—C5—N1	−163.1 (6)	N1—C5—C6—C8	−64.5 (8)
O4—C4—C5—N1	17.2 (8)	C4—C5—C6—C8	172.2 (6)

### Hydrogen-bond geometry (Å, º)

**Table:**

*D*—H···*A*	*D*—H	H···*A*	*D*···*A*	*D*—H···*A*
O6—H1*W*···O5^i^	0.85	1.85	2.693 (5)	175
O6—H2*W*···O5^ii^	0.85	1.88	2.723 (5)	175
N1—H1*N*···O3^iii^	0.90	2.15	2.979 (7)	153
N1—H2*N*···O1^iv^	0.90	2.41	3.103 (6)	133
C5—H5···O2^v^	0.99	2.59	3.527 (7)	158

Symmetry codes: (i) *x*−1, *y*, *z*; (ii) *x*−1/2, −*y*+1/2, −*z*+1; (iii) −*x*+1, *y*+1/2, −*z*+1/2; (iv) *x*, *y*+1, *z*; (v) *x*+1, *y*, *z*.
